# Exploring UK Knife crime and its associated factors: A content analysis of online newspapers

**DOI:** 10.3126/nje.v12i4.49994

**Published:** 2022-12-31

**Authors:** Divya Vinnakota, Q.M. Rahman, Brijesh Sathian, Ancy Chandrababu Mercy Bai, Nikulin Deividas, Maneesha-Varghese Pellissery, Sajna Kizhackanaly Abdul Kareem, Md. Rakibul Hasan, Ali Davod Parsa, Russell Kabir

**Affiliations:** 1 Department of Nursing and Public Health, University of Sunderland in London, UK; 2 NHS, London, UK; 3 Geriatrics and long-term care Department, Rumailah Hospital, Doha, Qatar; 4 School of Allied Health, Anglia Ruskin University, Essex, UK; 5 Copenhagen Business School, Denmark

**Keywords:** Knife crime, UK, Content Analysis, Newspaper, Online

## Abstract

Knife crime has become a common phrase used by the media, but it is not always clear what it refers to or what they mean when they use the term. Knife crime can cover many offences, making it challenging to define and estimate its prevalence. This review aimed to evaluate potential knife crimes in the UK from 2011 to 2021 and analyse the causes and risk factors associated with the crimes. Six UK online news portals were purposefully chosen to be included in the study, and knife crime news was searched retrospectively. The term “knife crime” was used to search. The news portals were the: Metro, the Sun, the Guardian, Daily Mail, Daily Mirror and the Evening Standard. In the assigned news portals, 692 reports were found between January 2011 and December 2021. The study revealed that the 11-20 years of age group individuals are more vulnerable as victims, and males are more reported as victims when compared to females. About 61.8% of knife crimes are reported from South England. Knife crime risk is higher in early adulthood and among males. Street violence, fights/gang attacks, family issues and robbery are the leading causes of knife crime and have all been identified as risk factors that must be addressed with caution.

## Background

Knife crime has become a common phrase used by the media, but it is not always clear what it refers to or what they mean when they use the term. Knife crime can cover many offences, making defining and estimating its prevalence challenging. Undoubtedly, producing a knife in the commission of a crime, such as a robbery or sexual assault, is a ‘knife crime,’ regardless of intent. It disproportionately impacts young individuals and people from disadvantaged backgrounds [1]. Additionally, the media referred to crimes committed with knives as an “epidemic” that significantly influenced younger generations [2]. This ‘epidemic’ resulted in the death of the victim, injuries to body parts or internal organs, fractures, scarring, and mental trauma[3].

Attacks with knives and fatal stabbings occur worldwide, even in the nations with the lowest overall crime rates and the highest violent crime rates [4]. According to the 2019 Global Study on Homicide published by the United Nations Office on Drugs and Crime, knives were the murder weapon of choice in 97,183 of the world’s total killings in 2017. This rate accounts for 22% of the total homicides [5]. Many “lone wolf” assailants turn to the kitchen knife as their preferred weapon for their crime. In addition, they use various sharp tools, such as scissors and axes [5]. The frequency of knife-related violence varies significantly from one region to the next.

Knife crime is linked to individual risk factors like gender [6]; age [7]; ethnicity, financial deprivation, and socio-economic background [8-9]; exposure to violence and prior victimisation [8]; mental illness and drug addiction [4]; low educational attainment and exclusion from mainstream education [10]. Family background and adverse childhood experiences [11]; lack of accessible alternative activities; gang involvement and territoriality [12]; deprivation, and violence [11] are also risk factors at the interpersonal and community level.

Knife crime offences in England and Wales have increased by 80% in the last five years [13], reaching levels not seen since 1946 [14]. Offences involving knives or sharp instruments rose by six per cent from 47,388 to 50,019 in England and Wales before the first Covid-19 lockdown was imposed [15]. Of these offences, 44% were for assault with injury or assault with intent to cause serious harm, and 44% were used in robbery [15]. Most of the victims and the perpetrators of these crimes were Black or Asian, especially young people aged 10 to 25 [16-18]. This gap could be related to the more significant proportion of BAME people living in London [16], which accounts for nearly a third of knife offences [17-18].

When unemployment rises, and work opportunities are limited, it may be difficult for some younger folks to see a promising future. They were forced to resort to risky means of obtaining money since no other option was available, and they carried knives to defend themselves. A comprehensive understanding of the causes and risk factors of knife crimes could prevent the increasing knife crime burden, especially among youth. Knife crime in the UK is considered a growing public health concern and in-depth knowledge of UK knife crime statistics are necessary. Until now, no such report has been published on knife crime in the UK. This review aimed to evaluate potential knife crimes in the UK from 2011 to 2021 and analyse the causes and risk factors associated with the crimes.

## Methods

### Data collection

Six UK online news portals were purposefully chosen to be included in the study, and knife crime news was searched retrospectively by seven authors. Because English is the country’s official language, only English online news portals were chosen. The portals were selected after the authors conducted a background search before beginning the study. The authors concentrated on the circularity of the portals and made conclusions based on their findings. The authors chose the most popular portals on this purpose. The term “knife crime” was used to search. The news portals were the: Metro, the Sun, the Guardian, Daily Mail, Daily Mirror and the Evening Standard. Retrospectively, news portals were initially searched with the search term. The contents of online journals were chosen because they were readily available and made a retrospective analysis of knife crimes easier. Age, place of knife crime, crime date, and other identifying variables were used to find repetitions. Duplications were defined as reports of the same knife crime in different portals and a total of 167 duplicate reports were excluded. The final data is entered into the software after removing the repetitions. A total of 692 knife crime reports were assessed and analysed by Statistical Package for Social Science (SPSS) version 26 and Microsoft excel version 2018 software.

### Inclusion of news

News of that was indicated as knife crime, and knife crime news bounded by the geographical area of the UK (especially England) were considered inclusion criteria.

### Variables

Age of the victim and the perpetrator, gender of the victim and the perpetrator, race of the victim and the perpetrator (if known), number of victims and perpetrators involved, date published, leading cause of crime (if known) and the location are considered as variables of the study.

### Permission

No formal ethical clearance was considered because the data only included previously published information available online.

## Results

In the assigned news portals, 692 reports were found between January 2011 and December 2021. The metro reported 34% of knife crime cases among the six online news portals screened, followed by the guardian (26.2%), evening standard (15.6%), daily mirror (15.5%), and the sun (8.8%). Six hundred and eighty-two cases were reported, with 61.8 percent coming from the south and 14.9% from the north. In total, 911 people were victims in these 692 reports. Around 81.8 percent of reports mention only one victim, while 18.2% mention multiple numbers of victims (ranging from 2-to 8 people) (**Table 1**).

Out of the 911 victims, 71.7% reported cases were less than 40 years of age, 72.8% were male, and 22.2% were female. The majority of the cases did not mention the race of the victims. Out of those reported, White/European/British were 5.8%, black/African were 5.8%, and 2.2% were Asians. The top three reasons for knife crime are 23.1% for street violence, 15% for fights/gang attacks, and 7.7% for family issues as reported in **Table 2**.

The total number of perpetrators involved in these 692 reports was 879 individuals. Multiples perpetrators are involved in 13.6% of cases (ranging from 2-to 8 people), while 56.5% of subjects report the involvement of a single perpetrator (56.5%). Out of all perpetrators, 66.2% were male, and 6.4% were females. Most perpetrators were under 40 years of age (92.5%). Most of the perpetrator’s race is not mentioned. Of those reported, about 5.2% were Black/African, 4.6% were White/European/British, and remaining 1.5% were Asians as shown in **Table 3**.

## Discussion

As knife crime is an underexplored issue, we aimed to look into knife crime variables between January 2011 and December 2021 based on online news portal reports retrospectively. The study revealed that 11-20 years of age group individuals are more vulnerable as victims, and males are more reported as victims when compared to females. About 61.8% of knife crimes are reported from South England. Street violence is the primary cause of knife crime compared to all other reasons. The more vulnerable age group among the perpetrators is also 11-20.

Males are reported as perpetrators than females. There was a downfall in reported knife crimes between 2011 and 2016. Since 2017, there has been an inclination in the number of reported cases (Figure 1). Overall, it has been found that young adult males are both victims and perpratrators of knife crime.

Good social skills, self-esteem, academic achievement, strong bonds with parents, positive peer groups, positive school attachment, community involvement, and access to social support are all factors that can help prevent young people from becoming violent. Reduced risk factors and increased protective factors have been shown to reduce youth violence. Social policy and long-term systematic approaches to addressing the root causes of violence can make the region safer.

Another factor contributing to young people’s attitudes toward criminal knife behavior is a lack of understanding of the legal and medical consequences of knife stabbing. Safety measures like workshops and group discussions among young people at an earlier age in schools about the danger of knives are helpful in tackling the problem to some extent.

Knife crime is a public health concern that can be prevented, and steps should be taken to safeguard people’s quality of life. A wide range of studies would be required to properly assess the problem and take the necessary steps to prevent it.

Knife crime is an underrated social issue in the UK.The study’s findings may provide a still picture of knife crime variables in news reporting aspects because only six online news portals were examined from January 2011 to December 2021. But to the best of the authors’ knowledge, it is the first online news analysis on knife crime in the UK. There are certain limitations as the authors scrutinised the published online news portals, so the data source is not strictly scientific. Furthermore, the study has used only six national newspaper and some important variables were missing. More broad studies would contribute to closing the large information gap in knife crime research in the United Kingdom.

## Conclusion

Knife crime has yet to gain attention in the United Kingdom. Knife crime risk is higher in early adulthood and among males. Street violence, fights/gang attacks, family issues, and robbery are the leading causes of knife crime and have all been identified as risk factors that must be addressed with caution. From January 2011 to December 2021, the knife crime rate appears to be rising from 2017, with the highest rate in 2018.

## Figures and Tables

**Table 1:** Distribution of demographic variables of the victims mentioned in 6 UK online news portals

Demographic variables	Frequency (n=911)	Percentage (%)
**Age in years**		
**Under 10**	23	2.5
**11-20**	376	41.3
**21-30**	154	16.9
**31-40**	100	11
**Over 40**	107	11.7
**Sex**		
**Male**	663	72.8
**Female**	202	22.2
**Race**		
**White/European/British**	53	5.8
**Black/African**	53	5.8
**Asian**	20	2.2
**American**	8	0.9
**Others**	7	0.8
**Location**	**n=692**	
**North England**	103	14.9
**South England**	428	61.8
**East England**	34	4.9
**West England**	31	4.5
**Central England**	76	11
**Others**	20	2.9

**Table 2:** Cause of the knife crimes mentioned in 6 UK online news portals

Cause of the knife crime	Frequency (n=692)	Percentage
**Family issues**	53	7.7
**Relationship issues**	34	4.9
**Mental health issues**	37	5.3
**Robbery**	41	5.9
**Fights/gang attacks**	104	15
**Street violence**	160	23.1
**Substance misuse related**	12	1.7
**Unprovoked**	33	4.8
**Not known**	107	15.5
**Others**	92	13.3

**Table 3:** Distribution of demographic variables of the perpetrators mentioned in 6 UK online news portals

Demographic variables	Frequency (n=879)	Percentage
**Age in years**		
**Under 10**	3	0.3
**11-20**	244	27.8
**21-30**	138	15.7
**31-40**	83	9.4
**Over 40**	66	7.5
**Sex**		
**Male**	582	66.2
**Female**	56	6.4
**Race**		
**White/European/British**	40	4.6
**Black/African**	46	5.2
**Asian**	13	1.5
**Others**	1	0.1
